# Rethinking TYK2 P1104A: a flawed evolutionary trade-off in tuberculosis?

**DOI:** 10.3389/fimmu.2026.1845279

**Published:** 2026-06-16

**Authors:** Pere-Joan Cardona

**Affiliations:** 1 Experimental Tuberculosis Unit (UTE), Institut de Recerca Germans Trias i Pujol (IGTP), Badalona, Spain; 2 Microbiology and Genetics Department, Universitat Autònoma de Barcelona, Bellaterra, Spain; 3 Servei de Microbiologia, Laboratori Clínic de la Metropolitana Nord (LCMN), Hospital Universitari Germans Trias i Pujol (HUGTiP), Badalona, Spain; 4 Centre de Medicina Comparativa i Bioimatge de Catalunya (CMCiB), Badalona, Spain; 5 Centro de Investigación Biomédica en Red de Enfermedades Respiratorias (CIBERES), Madrid, Spain

**Keywords:** evolutionary genetics, host–pathogen interactions, IL-12/IFN-γ axis, IL-23/IL-17 pathway, immune modulation, plague, tuberculosis, TYK2 P1104A

## Abstract

The TYK2 P1104A variant has been cast as a textbook example of evolutionary compromise-protecting against autoimmunity whilst increasing susceptibility to tuberculosis (TB). This narrative has been invoked to explain its decline in frequency in European populations since the Bronze Age. However, accumulating evidence suggests that this widely accepted trade-off may be more complex than originally proposed. At the mechanistic level, P1104A reduces TYK2 catalytic activity without abolishing cytokine signalling, owing to compensatory, non-hierarchical JAK activation. Critically, IL-12-dependent interferon-γ responses, central to antimycobacterial immunity, remain largely preserved. By contrast, IL-23 signalling is selectively impaired, leading to attenuated Th17 responses, and type I interferon signalling is also dampened. Rather than necessarily compromising host defence, this configuration could influence TB outcome by limiting IL-17-driven neutrophilic pathology and by curbing type I IFN-mediated suppression of protective Th1 immunity. Consistent with this model, clinical use of IL-23 and IL-17 inhibitors has not resulted in a meaningful rise in TB reactivation, further challenging the assumption that these pathways are essential for protection against TB in humans. Together, these observations support a more nuanced interpretation than a simple evolutionary trade-off. Instead, TYK2 P1104A may fine-tune immune responses by uncoupling protective Th1 immunity from deleterious inflammation. An additional speculative possibility is that such an immune configuration may also have influenced responses to other pathogens, including pneumonic plague caused by *Yersinia pestis*. Although speculative, this perspective suggests that multiple infectious pressures -not TB alone- may have shaped the evolutionary trajectory of this variant.

## Introduction

Boisson-Dupuis et al. (1) proposed that the TYK2 missense variant rs34536443, in which the minor C allele encodes the P1104A substitution, may have contributed to tuberculosis (TB)-driven negative selection in Bronze Age Europe. The aim of this perspective is to reassess that interpretation considering the broader functional literature on TYK2 signalling, autoimmunity, and host defence. Taken together, the available evidence suggests a more complex picture than a simple trade-off between protection from autoimmune disease and a marked increase in TB susceptibility.

## From increased to reduced risk of multiple sclerosis

Interest in rs34536443 intensified after a genome-wide association study in the Wellcome Trust Case-Control Consortium linked TYK2 to multiple sclerosis (MS) susceptibility (2). Subsequent analysis in a French MS case-control and trio-family cohort, however, reported the opposite direction of effect: the C allele was enriched in controls, indicating protection rather than risk (3). Although TYK2 mRNA and protein abundance were similar across genotypes in expanded T lymphocytes, stimulation with interferon-β (IFN-β) revealed reduced phosphorylation of TYK2 at residues Y1054/Y1055 in carriers of the P1104A allele, together with diminished induction of IFN-β-responsive genes such as MXA, OAS1, IRF1 and SOCS3. The variant also attenuated signalling downstream of IL-6 and IL-10, again consistent with reduced TYK2 activity (**Supplementary Table 1**).

Functionally, the variant was associated with a shift towards a Th2-like transcriptional profile, with increased GATA3, IL-13 and IL-5 expression, but without clear changes in Th1, Th17 or Treg markers, and without an obvious effect on the M1/M2 balance in peripheral blood monocytes. The authors concluded that the biological effect of the variant was modest, in line with its moderate protective effect against MS, and proposed that partial TYK2 hypofunction could represent a shared mechanism underlying protection across several autoimmune disorders (3).

## From non-hierarchical activation to gene dosage

**Figure 1:**
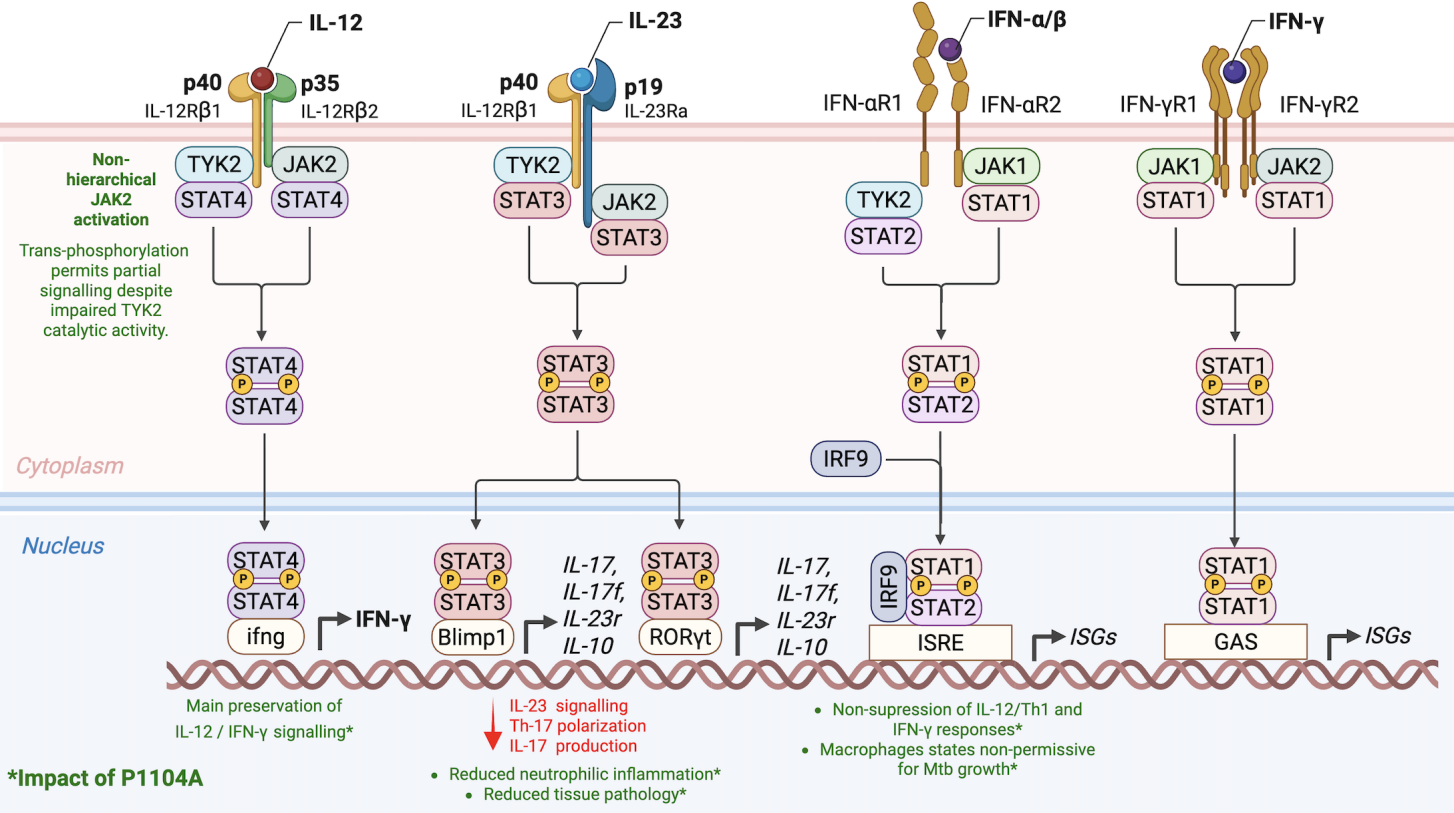
Differential effects of the TYK2 P1104A variant across IL-12, IL-23, type I IFN, and IFN-γ signalling pathways. Schematic representation of cytokine receptor signalling pathways affected by the TYK2 P1104A variant. Despite impaired TYK2 catalytic activity, IL-12 signalling retains function through non-hierarchical JAK2 trans-phosphorylation, preserving main STAT4-dependent IFN-γ responses. In contrast, the spatial and functional organization of the IL-23 receptor complex resembles that of the type I IFN receptor pathway, where TYK2-dependent receptor stabilization and signalling are more critical, resulting in preferential attenuation of IL-23/Th17 and IFN-I responses. The figure highlights the proposed consequence of this signalling asymmetry: reduced IL-17-driven inflammation and tissue pathology with relative preservation of IL-12/IFN-γ-mediated antimycobacterial immunity. Asterisks indicate proposed or context-dependent effects associated with P1104A. Made with Biorender.

Later functional studies in cytokine-responsive cell lines clarified why a catalytically impaired TYK2 variant can still support signalling. Experiments in TYK2-deficient, JAK1-deficient, and Epstein-Barr virus-transformed B-cell lines showed that P1104A is catalytically impaired but not signalling-dead. Because many cytokine receptors recruit two JAK kinases, one kinase can phosphorylate the other in trans. Under this non-hierarchical mode of activation, an inactive kinase can still stabilize the receptor complex and act as a scaffold whilst being phosphorylated by its partner. As a result, the consequence of a TYK2 mutation depends strongly on receptor architecture and on the identity of the associated JAK partner (**Figure 1**).

A subsequent multi-omic analysis of the TYK2 locus across autoimmune diseases further showed that rs34536443 is protective and, importantly, that its effect is non-additive: homozygosity confers substantially greater protection than heterozygosity. This dosage effect became a central theme in later work, both in human systems and in mouse models (4).

## Confirming a low-autoimmunity genotype

CRISPR-Cas9 engineering of rs34536443 into HEK293T cells confirmed reduced TYK2 and STAT phosphorylation after IFN-β stimulation, albeit to a lesser extent than complete TYK2 knockout. Likewise, immune cells from healthy Oxford BioBank donors carrying the homozygous variant showed attenuated STAT1 and STAT3 phosphorylation in response to type I interferons across all immune cell subsets. Responses to IL-12 and IL-23 were also reduced, whereas signalling downstream of IL-6, IL-10 and IL-13 was largely preserved. These findings were mirrored functionally by reduced Th1 and Th17 responses, with no effects in Th2 cytokine production (4).

Mouse models carrying the orthologous P1124A substitution yielded a similar picture. Type I IFN-, IL-12- and IL-23-induced TYK2 phosphorylation was impaired, yet the overall immune phenotype remained close to that of wild-type animals. In experimental autoimmune encephalomyelitis, heterozygous mice showed reduced disease incidence and homozygous animals were completely protected, consistent with a marked reduction in pathogenic Th1 and Th17 responses. Collectively, these data support the view that P1104A lowers autoimmune risk by partially dampening TYK2-dependent inflammatory pathways rather than by producing clear immunodeficiency (4).

## Relating TYK2 P1104A to *Mycobacterium tuberculosis* infection

Analysis of 116,732 genotyped participants from the UK Biobank, including 249 carriers of the homozygous genotype, did not show increased hospitalization for mycobacterial, bacterial, viral or fungal infections. This observation suggested that the residual cytokine signalling preserved by P1104A is sufficient to maintain broad antimicrobial defence, whilst still limiting autoimmune pathology (4).

Against this background, current understanding of TB immunopathogenesis is particularly relevant (5). IL-12 is a central protective cytokine because it drives Th1 differentiation and IFN-γ production, thereby activating macrophages to control intracellular bacilli. IL-23, which shares the p40 subunit with IL-12, has a more prominent role in early inflammatory amplification through IL-17-producing cells. IL-17 promotes recruitment of neutrophils and other leukocytes, especially under conditions of high bacillary burden, a scenario favoured in the upper pulmonary lobes (6). IL-6 reinforces this inflammatory circuit by promoting T-cell activation and Th17 differentiation. By contrast, IL-10 dampens protective immunity, and type I interferons can worsen TB by suppressing anti-mycobacterial responses and favouring macrophage states that are more permissive for bacterial growth (7, 8).

This balance matters because excessive neutrophilic inflammation is increasingly recognized as a hallmark of progression towards active pulmonary TB. Once lesions become dominated by extracellular bacillary growth and tissue-destructive inflammation, granuloma containment can fail (6, 9, 10). In that context, a genotype that partially reduces IL-23/IL-17-driven pathology whilst preserving substantial IL-12-dependent IFN-γ responses might not necessarily increase susceptibility to progression towards active disease and could potentially modulate inflammatory pathology.

A further consideration concerns the role of IL-23 in long-term Mtb containment. Beyond its function in early inflammatory amplification, IL-23 is required for the CXCL13-dependent formation and maintenance of pulmonary B-cell follicles and inducible bronchus-associated lymphoid tissue (iBALT), structures increasingly regarded as morphological correlates of protective immunity. IL-23-deficient (Il23a−/−) mice show increased bacterial burden late in infection, smaller and disorganised B-cell follicles, and reduced CXCL13 expression, whereas type-1 (IFN-γ) responses remain largely intact (11, 12). Because P1104A attenuates rather than abolishes IL-23 signalling, and because follicle/iBALT formation is redundantly supported by several stromal-activating cytokines (type I IFN, IL-1, IL-17, IL-22 and IL-13), a partial reduction in IL-23 output need not recapitulate the follicle failure seen with complete IL-23 ablation. Consistent with this, IL-17RA- and IL-22-deficient mice — lacking the principal effector arms downstream of IL-23 — control Mtb as efficiently as wild-type animals, and neither cytokine accounts for the late defect of Il23a−/− mice (11). Nonetheless, the possibility that lifelong, gene-dosage-dependent attenuation of IL-23 in P1104A homozygotes subtly impairs pulmonary follicle organisation over decades of chronic exposure cannot be excluded, and merits dedicated investigation; this represents an important caveat to the model proposed here.

## Bronze Age selection and the tuberculosis hypothesis

Boisson-Dupuis et al. (1) investigated TYK2 P1104A in patients with Mendelian susceptibility to mycobacterial disease and paediatric or early-onset TB. Across 3,752 exomes, they identified 168 heterozygotes and 11 homozygotes; seven of the homozygotes had TB and three had Mendelian susceptibility to mycobacterial disease. After adjustment for ancestry by principal-component analysis, homozygosity appeared enriched in affected individuals, whereas heterozygosity did not. Based on these findings and ancient DNA data, the authors proposed that P1104A may have undergone negative selection in Europe because it increased vulnerability to TB.

In the largest available ancient DNA analysis of 230 ancient Eurasians (13), the authors identified the P1104A variant in central European individuals, observing a marked decline in its frequency from approximately 9% in the late Neolithic period (around 4000 BC), to 4,2% by 300 BC. After evaluating different hypotheses, they proposed that the P1104A was likely purged in Europe by TB since the Neolithic. This is because no other intramacrophagic infection, whose control depends on IFN-γ, has been endemic in Europe for such an extended period with the capacity to cause mortality during childhood or reproductive age (14), making this a plausible example of negative selection (15).

Their functional work also provided important mechanistic insights. In contrast to earlier interpretations, signalling through IL-12 remained largely intact in P1104A homozygotes, whereas IL-23 signalling was severely compromised. Experiments in reconstituted cell systems suggested that IL-12 signalling can proceed when only one of the two associated kinases is catalytically active, whereas IL-23 signalling requires both kinases to be active (**Figure 1**).

This difference can be explained by the receptor architecture: although the IL-12 and IL-23 pathways share a receptor chain (IL-12Rβ1) and two kinases (TYK2 and JAK2), JAK2 binds to IL-23R at a site located much farther from the juxtamembrane region than in the IL-12Rβ2 complex. This structural arrangement likely accounts for the impaired IL-23-dependent IFN-γ production, whilst IL-12-dependent Th1 differentiation and IFN-γ production remain largely preserved (1).

Peripheral blood mononuclear cells from homozygous individuals confirmed this dissociation. Responses to IL-12 were normal in whole blood and PBMC assays, whereas responses to IL-23 were defective, with reduced IL-17A, IL-17F and IL-22 production by memory CD4-positive T cells. Notably, despite impaired IL-17 immunity, these individuals do not show a clear syndrome of chronic mucocutaneous candidiasis, underscoring that the phenotype is selective and more subtle than complete pathway deficiency (1).

## IL-12 impairment appears dosage-dependent rather than absolute

Subsequent work by Gorman et al. (16) reinforced the data of Dendrou et al. (4). Using the murine orthologue P1124A, the authors found that homozygous cells showed diminished STAT phosphorylation after IL-12 stimulation and reduced capacity of CD4-positive T cells to skew towards a Th1 phenotype *in vitro*, whereas heterozygous cells behaved much more like wild type. In both mice and human PBMCs, several TYK2-dependent phenotypes displayed a clear gene-dosage effect, with heterozygotes showing intermediate signalling defects and homozygotes showing the strongest phenotype.

The same study also reported reduced frequencies of T follicular helper cells and memory germinal-centre B cells in healthy human carriers, again without evidence of severe immune collapse. In experimental autoimmune encephalomyelitis, homozygous mice remained protected, and this correlated with reduced frequencies of IFN-γ-positive and IFN-γ/IL-17 double-positive CD4-positive T cells in draining lymph nodes and in the central nervous system. These findings further support the idea that P1104A primarily reshapes inflammatory set-points rather than simply weakening host defence.

## Rise and fall of P1104A in West Eurasia: an ecological interpretation

Ancient-DNA studies extended the story beyond clinical immunology. Analyses of European genomes from the Mesolithic to the Middle Ages (17) showed that P1104A first appeared around 6500 BC. in Anatolia, rose to appreciable frequencies during the Bronze Age (10%), to decline by the Iron Age to its current low frequency (2.9%). The Bronze Age was also a period of major demographic transition, marked by the Yamnaya or Pit Grave culture from the Pontic Steppe region (18, 19), strong sex-biased migration (20), Y-chromosome bottlenecks (21) and the spread of novel pathogens, including a non-flea adapted *Yersinia pestis* lineage causing pneumonic plague (22, 23).

It has been proposed that the dissemination of this highly lethal disease (mortality up to 100%), may have facilitated the male dominance of a small group of migrants over larger, already organized central European farmers, including those associated with the Cucuteni-Trypillia mega-settlements (24).

This scenario is reminiscent of the rapid conquest of large and complex pre-Columbian American empires by relatively few conquistadors in the 15th century, a process largely attributed to the introduction of novel and virulent pathogens such as variola, influenza and measles (25). In this context, it is conceivable that P1104A may have influenced inflammatory responses in Pontic Steppe migrants, during chronic TB infection (26) in ways that indirectly affected susceptibility to other pathogens. One speculative possibility is that persistent Th1-skewed immune activation associated to latent or subclinical TB infection could have modified host responses to pneumonic infections such as *Yersinia pestis* (27). Importantly, no direct genetic, epidemiological, or functional evidence currently links TYK2 P1104A itself to altered susceptibility to *Yersinia pestis*, and this interpretation should therefore be considered hypothetical.

Moreover, recent evidence indicates that IL-17 induction during pneumonic plague promotes neutrophil recruitment and sustains neutrophilic inflammation. Notably, similar features are observed in *Mycobacterium tuberculosis* infection, where both pathogens evade neutrophil antibacterial activity, facilitating extracellular bacillary growth and contributing to disease pathology (28).

## Anti-IL-23 and anti-IL-17 therapies do not support a strong tuberculosis-risk model

A clinically relevant line of evidence comes from modern immunomodulatory therapy. In experimental TB models, IL-23 deficiency or IL-23 blockade does not markedly compromise control of mycobacterial infection or immune memory to BCG. Likewise, IL-17 blockade had little effect on bacterial burden in the murine model (29). Consistent with this, growing clinical experience with IL-23 and IL-17 inhibitors in psoriasis has not revealed a major signal for TB reactivation, even amongst patients with latent TB infection who did not receive chemoprophylaxis (30).

Recent retrospective multicentre studies and systematic reviews report very low TB reactivation rates in patients treated with IL-17 or IL-23 inhibitors (31). Nevertheless, pharmacologic cytokine blockade does not fully recapitulate lifelong germline TYK2 dysfunction. Biologic therapies are typically initiated in adulthood, are time-limited, and may not reproduce developmental, compensatory, or gene-dosage effects associated with inherited TYK2 variants. Therefore, these observations should be interpreted cautiously and viewed as indirect evidence arguing against a major TB susceptibility phenotype driven solely by selective IL-23/IL-17 impairment.

A recent population-based analysis of the TriNetX network reported a two- to five-fold higher crude TB incidence for IL-17, IL-23 and IL-12/23 inhibitors relative to the general population (32). These crude estimates should be interpreted with caution: TB was ascertained from a single unvalidated ICD-10 range, the cohort’s baseline TB incidence (≈22 per 100,000) exceeded the national estimate (≈3 per 100,000) by nearly an order of magnitude, intensive guideline-mandated screening of psoriasis patients introduces confounding by surveillance, and platform-level rounding of small event counts can inflate rare-event associations (33). Notably, in the same study’s multivariable models, all interleukin-pathway inhibitors carried a lower adjusted TB hazard than TNF-α inhibitors (adjusted hazard ratios 0.39–0.54). Accordingly, current expert consensus advises against routine latent-TB testing prior to or during IL-17/IL-23 inhibitor therapy (34). Taken together, the available clinical evidence indicates that selective IL-23/IL-17 blockade carries, at most, a modest TB-reactivation risk — considerably lower than that of TNF-α inhibition — rather than the marked susceptibility predicted by a simple essential-pathway model.

## Reduced type I IFN signalling via P1104A could represent an additional protective mechanism against TB

A second, mechanistically independent consequence of P1104A deserves emphasis. Type I interferons are increasingly recognised as detrimental rather than protective in TB: they antagonise IL-12-driven Th1 differentiation and IFN-γ responses, promote macrophage states permissive for intracellular bacillary growth, and accompany progression to active disease (7, 8). Because TYK2 is constitutively associated with IFNAR1 and is the dominant kinase initiating type I IFN signalling, P1104A attenuates this axis directly indeed, reduced type I IFN-induced STAT phosphorylation is amongst the most consistent functional signatures of the variant across human and murine systems (3, 4). Dampening a pathway that suppresses protective Th1 immunity and favours bacterial persistence may therefore constitute a protective effect of P1104A that is distinct from, and potentially stronger than, its attenuation of IL-23/IL-17-driven inflammation (**Figure 1**). This further argues against a simple model in which P1104A increases TB susceptibility.

## A more plausible interpretation

At a population level, TB risk is driven by a complex mixture of undernutrition, diabetes, smoking, alcohol use, HIV infection (35), exposure intensity, reinfection, socio-environmental conditions and host genetics (36, 37). In that setting, the contribution of any single common variant is likely to depend on genetic background, pathogen background and environmental context. Recent reviews have therefore emphasized that the genetic architecture of TB susceptibility is highly polygenic and remains difficult to capture even with large genome-wide studies (38).

**Figure 2:**
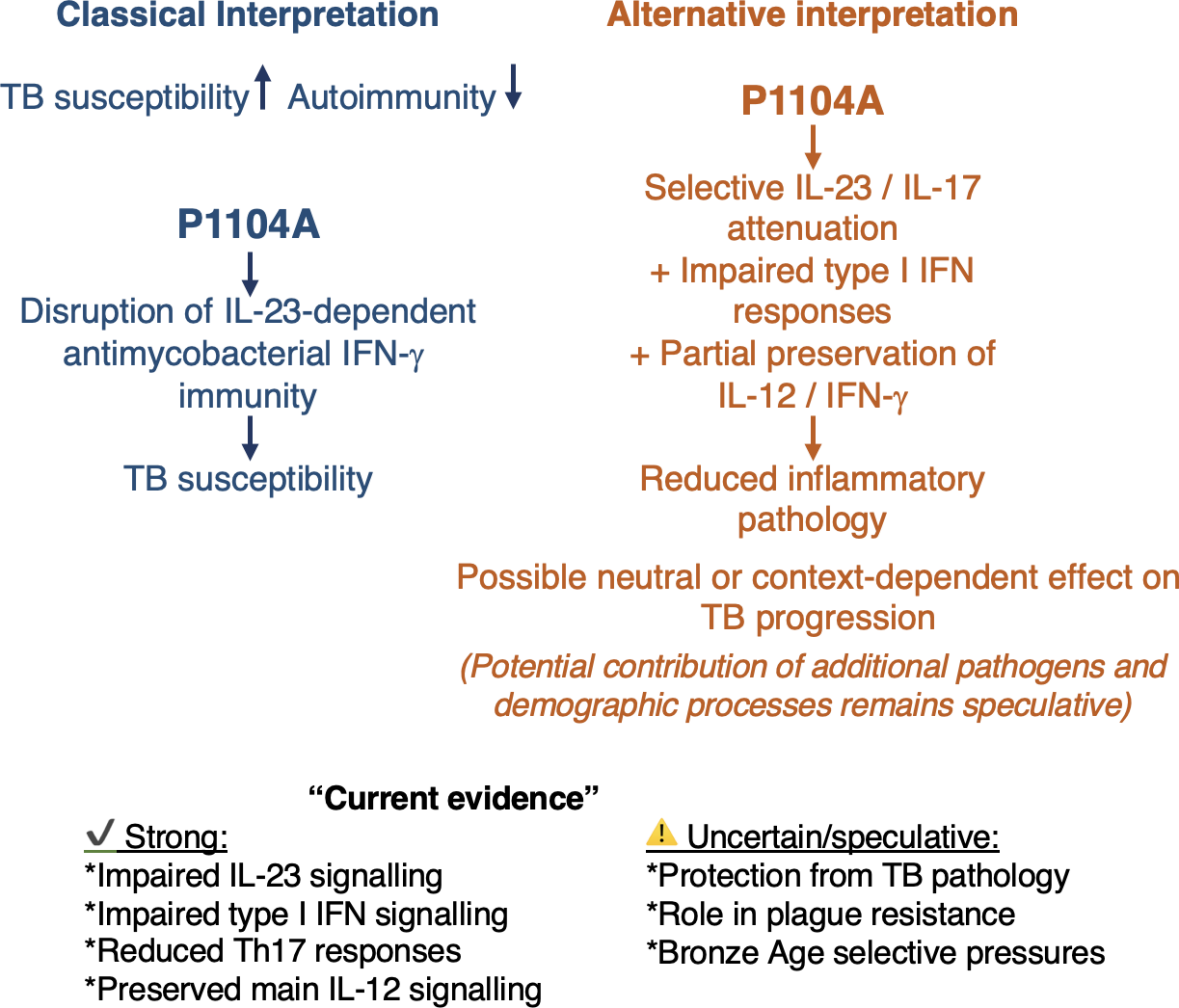
Conceptual models for the immunological and evolutionary impact of the TYK2 P1104A variant. The classical model links P1104A to increased tuberculosis (TB) susceptibility through impaired IL-23–dependent IFN-γ immunity. An alternative model proposes that the variant preferentially dampens IL-23/Th17 and type I IFN pathways whilst preserving main IL-12/IFN-γ responses, potentially reducing inflammatory pathology with neutral or context-dependent effects on TB progression. The figure also distinguishes evidence currently supported by experimental data from broader evolutionary hypotheses that remain speculative.

Viewed in this broader framework, TYK2 P1104A does not appear to be a compelling example of a clean evolutionary trade-off in which protection from autoimmunity is paid for by a clear increase in active TB (39). Instead, the available data are compatible with a model in which the variant dampens type I IFN and IL-23/IL-17 signalling, thereby reducing immunopathology, whilst preserving enough IL-12-dependent Th1 immunity to maintain control of mycobacterial infection in most contexts (**Figure 2**).

## Conclusion

Overall, the literature points to TYK2 P1104A as a partial loss-of-function variant that recalibrates inflammatory immunity rather than abolishing it. By attenuating type I IFN and IL-23/IL-17 pathways, the variant may reduce tissue-damaging inflammatory responses associated with autoimmunity and, potentially, with progression from controlled infection to active TB. Dampened type I IFN signalling may be especially relevant, since this pathway suppresses IL-12-driven Th1 immunity and favours macrophage states permissive for bacterial growth during *Mycobacterium tuberculosis* infection; its attenuation could therefore constitute a protective effect distinct from, and potentially as important as, reduced IL-23/IL-17 inflammation. At the same time, non-hierarchical JAK activation helps preserve IL-12-dependent signalling and Th1-mediated IFN-γ production, which remain central to anti-mycobacterial defence.

These potential benefits should be weighed against a plausible cost. IL-23 contributes to CXCL13-dependent B-cell follicle and inducible bronchus-associated lymphoid tissue (iBALT) formation and to long-term mycobacterial containment. Because P1104A attenuates rather than abolishes IL-23 signalling, and because follicle formation is redundantly supported by several stromal-activating cytokines, this cost is likely partial; nonetheless, the long-term consequences of lifelong IL-23 attenuation for pulmonary follicle organisation warrant dedicated investigation.

Taken together, the available data do not yet support a simple model in which TYK2 P1104A acted as a major and isolated determinant of TB susceptibility or negative selection in Bronze Age Europe. A more cautious interpretation is that the variant may sit at an immunological intersection between reduced inflammatory pathology and largely retained host defence, with its evolutionary trajectory shaped not only by infection but also by demography, ecology, and the broader pathogen landscape.

## Data Availability

The original contributions presented in the study are included in the article/**Supplementary Material**. Further inquiries can be directed to the corresponding author.
